# Synthesis and Characterization of 2D Ternary Compound TMD Materials Ta_3_VSe_8_

**DOI:** 10.3390/mi15050591

**Published:** 2024-04-28

**Authors:** Yuanji Ma, Yuhan Du, Wenbin Wu, Zeping Shi, Xianghao Meng, Xiang Yuan

**Affiliations:** 1 State Key Laboratory of Precision Spectroscopy, East China Normal University, Shanghai 200241, China; 2 Shanghai Center of Brain-Inspired Intelligent Materials and Devices, Department of Electronics, East China Normal University, Shanghai 200241, China

**Keywords:** Ta_3_VSe_8_, 2D materials, ternary compound TMDs, synthesis

## Abstract

Two-dimensional (2D) transition metal dichalcogenides (TMDs) are garnering considerable scientific interest, prompting discussion regarding their prospective applications in the fields of nanoelectronics and spintronics while also fueling groundbreaking discoveries in phenomena such as the fractional quantum anomalous Hall effect (FQAHE) and exciton dynamics. The abundance of binary compound TMDs, such as MX_2_ (M = Mo, W; X = S, Se, Te), has unlocked myriad avenues of exploration. However, the exploration of ternary compound TMDs remains relatively limited, with notable examples being Ta_2_NiS_5_ and Ta_2_NiSe_5_. In this study, we report the synthesis of a new 2D ternary compound TMD materials, Ta_3_VSe_8_, employing the chemical vapor transport (CVT) method. The as-grown bulk crystal is shiny and can be easily exfoliated. The crystal quality and structure are verified by X-ray diffraction (XRD), while the surface morphology, stoichiometric ratio, and uniformity are determined by scanning electron microscopy (SEM). Although the phonon property is found stable at different temperatures, magneto-resistivity evolves. These findings provide a possible approach for the realization and exploration of ternary compound TMDs.

## 1. Introduction

Transition metal dichalcogenides (TMDs) stand as a significant family within the field of materials science, garnering widespread interest across various scientific domains [1,2,3]. They form a layered structure, and the transition metal atom layers are generally sandwiched between the chalcogen atom layers. They exhibit a van der Waals structure [4,5,6,7,8,9] similar to graphite, highlighting their potential for exfoliation. TMDs boast an array of distinctive properties, including an adjustable band gap [10,11], strong light-matter interactions [12,13], spin-orbit coupling [14,15,16], and valley-selective responses [17,18,19]. Owing to this wealth of unique attributes, TMDs are deemed promising candidates for a multitude of applications, including electronics, optoelectronics, thermoelectrics, nano-mechanics, and valleytronics.

Prior investigations have predominantly concentrated on binary compound TMDs. Notable findings include the observation of fractional quantum anomalous Hall effect (FQAHE) in twisted MoTe_2_ [20,21]. Charge density wave transition has been identified in 1T-TaS_2_ [22] and 2H-NbSe_2_ [23]. Superconductivity has been detected in 2H-TaS_2_ [24], 2H-WS_2_ [25,26,27], 2H-MoTe_2_ [26,27], 2H-MoS_2_ [26], and 1T-NbSeTe [28]. The exciton physics [29,30,31,32] of MoS_2_, MoSe_2_, WS_2_, and WSe_2_ have been extensively explored as well. Defect and chemical engineering [33,34] in MoS_2_, as well as strain engineering [35] in WSe_2_ and WS_2_, also have piqued considerable interest. However, studies regarding ternary compound TMDs (with two metal elements confined in transition metal) are comparatively scarce. Ta_2_NiSe_5_, for instance, has been identified as an excitonic insulator [36,37,38,39]. Photodetectors [40,41,42,43,44] fabricated based on Ta_2_NiS_5_ and Ta_2_NiSe_5_ present unique optoelectronic responses. The introduction of a new ternary compound TMD may broaden the horizons of existing research on TMDs. For instance, ternary compound TMDs offer supplementary parameters to modulate a sample’s characteristics, and, specifically, they can impact comprehensive properties by altering the elements’ proportion [45,46]. Additionally, ternary TMDs containing magnetic elements may even make the material itself naturally valley-polarized, thereby making a contribution to the research of valley electronics [47,48,49,50].

In this work, we report the synthesization of a new ternary compound TMD, Ta_3_VSe_8_. The single crystals are grown by chemical vapor transport (CVT) in a dual-zone furnace. We harvest the crystals from the colder end of the ampoule, with their sizes ranging from about 9 to 25 mm^2^. X-ray diffraction (XRD) and energy dispersive X-ray spectroscopy (EDX) analysis are conducted on Ta_3_VSe_8_ to ascertain the crystal quality and the homogeneity of its elemental distribution. Terrace features are witnessed on the surface using both optical microscopy and scanning electron microscopy (SEM). Through temperature-dependent Raman spectroscopy, we documented stable phonon vibrations across a series of temperatures ranging from liquid nitrogen temperature to room temperature. Furthermore, positive magnetoresistance is demonstrated around liquid helium temperature and becomes gradually suppressed at higher temperatures. Our findings on a new ternary compound TMD provide a possible playground in multi-element TMD research areas.

## 2. Materials and Methods

### 2.1. Sample Synthesis

**Figure 1:**
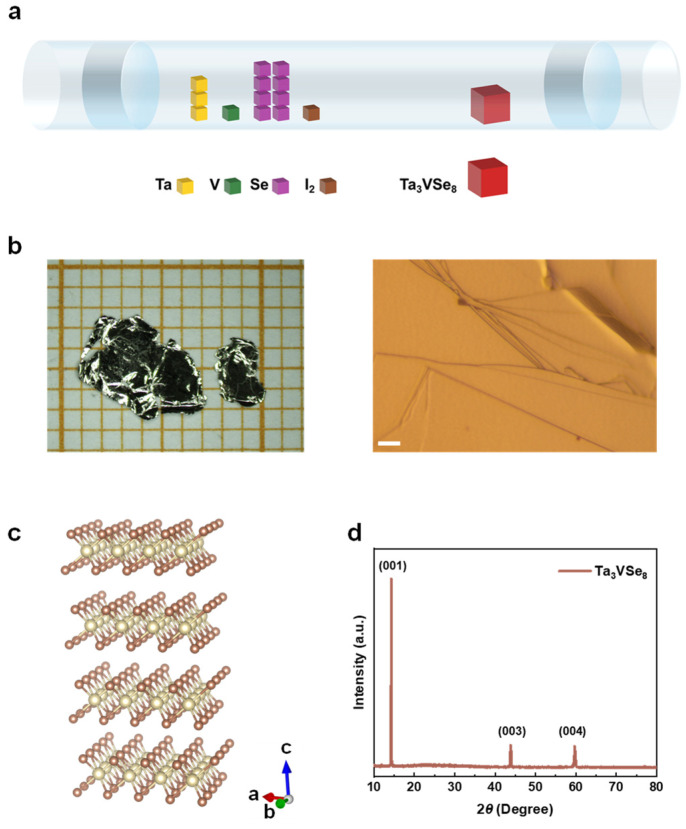
The synthesis and characterization of Ta_3_VSe_8_. (**a**) Schematic plot of the growth configuration. Starting materials are loaded in an evacuated ampule according to chemical ratios. The ampule is then placed in a dual-zone furnace with Iodine as a transport agent. (**b**) Image of single crystals. Left panel: Stereo-microscopic images. The size of the sample is 9 to 25 mm^2^. The unit square of the millimeter graph paper is 1 × 1 mm in size. Right panel: Metallurgical microscopic images. Terrace features are presented in the sample surface. The scale bar is 20 μm. (**c**) Schematic of the crystal structure. The structure belongs to the P-3m1 (No. 164) space group. The beige and brown balls represent the Ta (V) and Se atoms, respectively. (**d**) X-ray diffraction (XRD) pattern of Ta_3_VSe_8_. The diffraction peaks at 14.3°, 43.9°, and 59.7° are indicative of the (001) crystallographic plane, revealing the lattice constant along the *c*-axis to be 6.18 Å.

Due to the interesting properties founded in TaSe_2_ [51,52] and VSe_2_ [53,54,55], we synthesized the Ta_3_VSe_8_ flakes using chemical vapor transport (CVT) and by employing Tantalum (Ta) powders, Vanadium (V) powders, and Selenium (Se) shots (Figure 1a). Stoichiometric mixtures of the raw materials were sealed in quartz tubes under an argon (Ar) atmosphere. The Iodine (I_2_) shots were added as the transport agent to facilitate the synthesis process. Subsequently, the sealed ampoules were evacuated and strategically positioned into a dual-zone furnace with a temperature gradient of 200 K (1223 K at high-temperature zone and 1023 K at low-temperature zone). Following a week-long controlled cooling process, single crystals were harvested from the cold zone of the ampoules.

### 2.2. Sample Characterization

**Figure 2:**
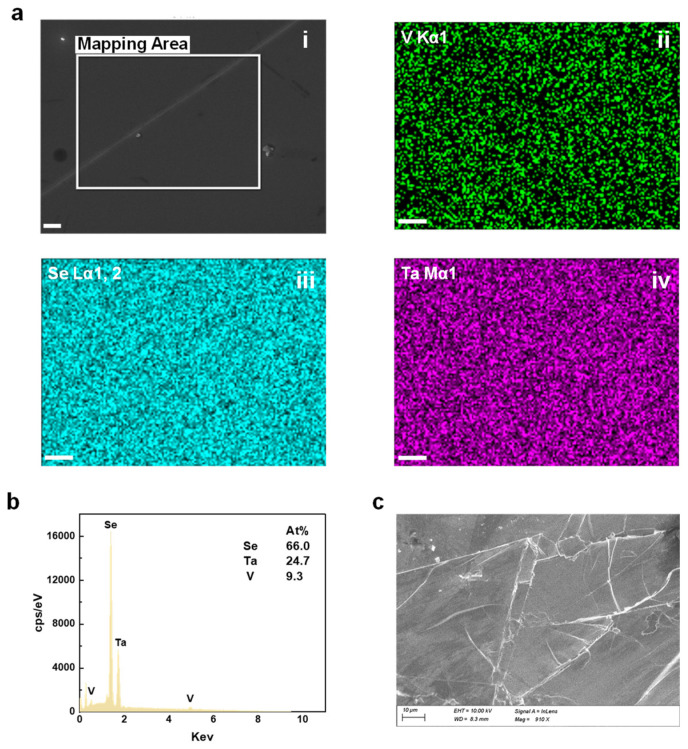
Energy Dispersive X-ray Spectroscopy (EDX) analysis. (**a**) Elemental mapping corresponding to the designated area (white square) (i). The distribution of, Vanadium (V), Selenium (Se), and Tantalum (Ta) is depicted in green (ii), blue (iii), and purple (iv), respectively. The scale bar is 2 μm. It points out the homogeneity within the Ta_3_VSe_8_ composition. (**b**) EDX results derived from the elemental mapping. The observed chemical ratio of the crystal is Ta:V:Se = 24.7:9.3:66.0, which aligns with the expected stoichiometry. (**c**) Scanning electron microscopy (SEM) photo of the same crystal in (**a**). The scale bar is 10 μm. The photo reveals the presence of terrace features on the surface of the sample.

**Figure 3:**
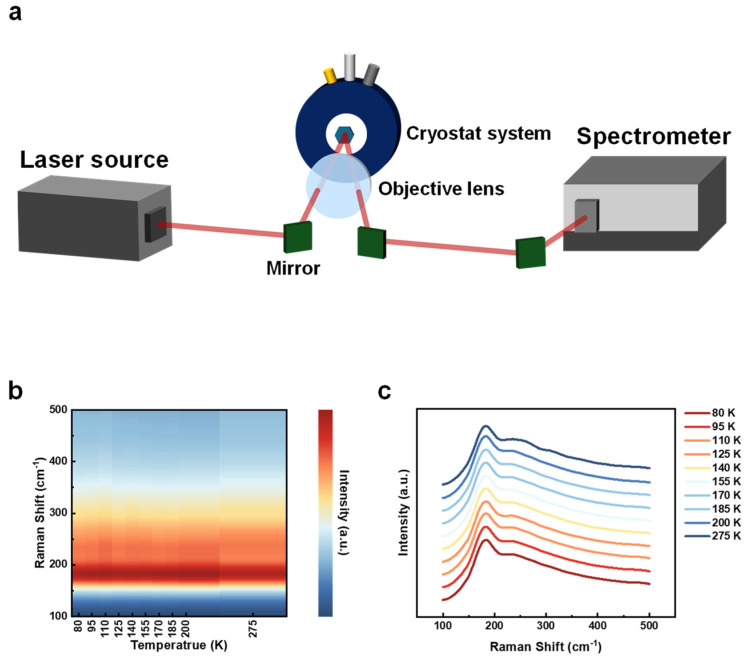
Temperature-dependent Raman spectroscopy. (**a**) The schematic of the Raman spectroscopy experimental setup. The excitation beam (λ = 632.8 nm) is generated by a helium-neon (He-Ne) laser source and is subsequently focused on the Ta_3_VSe_8_ through an objective lens. The scattering light is then harvested by the same lens and collected by the spectrometer directly. To realize the temperature control during the experiment, the sample is mounted in the cryostat system carefully. (**b**) False-color pattern of temperature-dependent Raman spectra. (**c**) Stacking plot of Raman spectra at different temperatures. According to (**b**,**c**), the Raman peaks of Ta_3_VSe_8_ illustrate stability as the temperature changes.

Energy dispersive X-ray spectroscopy (EDX), based on a scanning electron microscope (SEM, Zeiss GeminiSEM450) [56,57], was employed to determine the elemental composition of the Ta_3_Vse_8_ flakes. The elemental mapping (Figure 2a) validated the chemical ratio and homogeneous distribution within the crystals. X-ray diffraction analysis (Malvern Panalytical Empyrean) was performed to ascertain the crystallographic information of Ta_3_VSe_8_, confirming that Ta_3_VSe_8_ belongs to the P-3m1 (No. 164) space group. We studied the phonon and electron properties due to their importance in determining the crystal property in condensed matter. For temperature-dependent Raman spectroscopic experiments, we utilized a home-built optical setup (Figure 3a) integrated with a grating spectrometer (Oxford Andor Shamrock 500i). A helium-neon (He-Ne) laser beam (Thorlabs HNL225R) with a wavelength of 632.8 nm served as the excitation source. The beam was focused on the sample via an objective lens, and the scattered light was subsequently collected through the same lens. The light then flowed into the grating spectrometer to form Raman spectra. Critical to our temperature-dependent analysis, the sample was placed in a nitrogen-cooled cryostat system (Oxford MicrostatN) to realize temperature control from liquid nitrogen temperature to room temperature. 

### 2.3. Magneto-Transport Measurement

**Figure 4:**
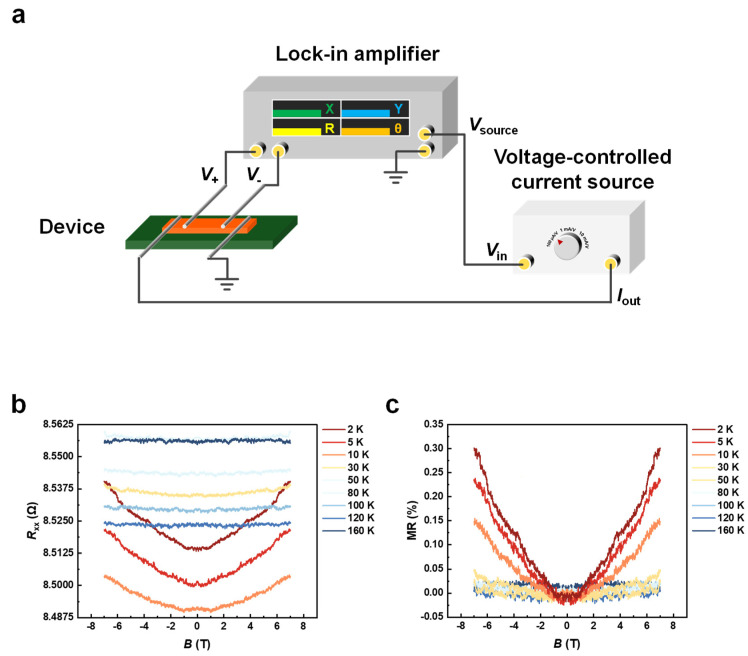
Magneto-transport measurement. (**a**) Experimental setup of transport measurement. (**b**,**c**) Temperature-dependent Magnetoresistance. We perform measurements across a range of temperatures extending from 2 K to over 100 K, as represented by a gradient of colors from red to blue in the plot. From this plot, we observe that Ta_3_VSe_8_ demonstrates a mildly positive magnetoresistivity at low temperatures and becomes suppressed at higher temperatures.

The sample was affixed to a DIP16 sample holder using GE varnish, followed by a connection between the sample and the electrodes using silver wires. The device was then incorporated into a commercial sample probe. The measurement was conducted in a superconducting magnet. Data acquisition was undertaken by a lock-in amplifier (Stanford SR860) paired with a voltage-controlled current source (Stanford CS580), ensuring a precise collection of magnetoresistance. We executed a series of magnetoresistance measurements, spanning a broad temperature range (Figure 4). 

## 3. Results and Discussion

Utilizing CVT growth with the precise chemical ratios of raw materials (Figure 1a), we successfully produce shiny single crystals at the low-temperature end of the ampule. As depicted in the figure, the vibrant yellow, green, and purple cubes symbolize the three raw materials: Tantalum (Ta) powders, Vanadium (V) powders, and Selenium (Se) shots, respectively. The proportional relationship between these cubes visually emulates the actual stoichiometric balance, Ta:V:Se = 3:1:8. The brown cubes represent the transport agent Iodine (I_2_), which is essential for the growth process (about 5 mg/cm^3^). The sizable red cube stands for the product, Ta_3_VSe_8_. Preliminary examination of the samples under a stereomicroscope (referenced in Figure 1b, left panel) confirms their shiny surfaces and substantial sizes, ranging from 9 to 25 mm², affirming their robust growth and suitability for future experimentation. To closely inspect the sample surface, we resort to a metallurgical microscope (Figure 1b, right panel). Terrace features are presented on the sample’s surface, which are further confirmed by scanning electron microscopy in Figure 2c. The structure of the crystal is constructed by Visualization for Electronic and STructural Analysis (VESTA) [58] in Figure 1c, adhering to the P-3m1 space group. Within this arrangement, Ta (V) and Se atoms are represented by spheres of differing colors, which are labeled beige and brown, respectively. Subsequently, we perform the X-ray diffraction analysis on Ta_3_VSe_8_ to evidence its well-grown nature and determine its lattice constant. The diffraction peaks are located at 2θ = 14.3°, 43.9°, and 59.7°. On the one hand, the distinct (00n) peak observed in the diffraction pattern attests to the crystalline state of the samples. On the other hand, combined with the wavelength of Cu Kα (*λ* = 1.5418 Å) and the peak positions, we calculated the lattice constant (d) along *c*-axis to be approximately $d=\frac{n\lambda }{2\sin\theta }$ = 6.18 Å.

To ascertain the actual elemental composition of the sample, we use EDX elemental mapping based on SEM. The elemental mapping, focusing on the area demarcated by the white square (i), is exhibited in Figure 2a. The distribution of, Vanadium (V), Selenium (Se), and Tantalum (Ta) is depicted in green (ii), blue (iii), and purple (iv), respectively. The nearly uniform spread of these colors across the mapping area demonstrates the homogeneity of the sample composition. The EDX results extracted from the mapping highlight the actual chemical stoichiometry within the sample as Ta:V:Se = 24.7:9.3:66.0. Judging from these results, we confirm that the crystal used in our experiment maintains an elemental ratio that mirrors the expectations. Meanwhile, we capture an SEM image (Figure 2c) of the crystal, which confirms the terrace features previously observed with the metallurgical microscope (see Figure 1b, right panel).

In an effort to delve deeper into the phonon dynamics of Ta_3_VSe_8_, we conduct temperature-dependent Raman spectroscopy utilizing a home-built optical system, as illustrated in Figure 3a. A He-Ne laser is chosen as the excitation laser source, which emits a beam with a wavelength of 632.8 nm. This beam is initially reflected by a mirror before being focused onto the sample surface via an objective lens. The lens not only directs the light toward the sample but also collects the scattered light, which encompasses the information regarding the phonon dynamics. Eventually, the light enters the grating spectrometer for analysis. To control the experimental temperature precisely, a cryostat system is used. The sample is securely affixed onto the system’s holder, and the cryostat features a glass window transparent to the He-Ne laser beam. The experimental temperature is continuously set between liquid nitrogen temperature and room temperature. The Raman spectroscopic results, graphically represented in Figure 3b,c, are displayed both as a false-color map and a stacking plot. Two prominent peaks of around 182 cm^−1^ and 234 cm^−1^ stand out in the spectra. As observed in both representations, these peak intensities and positions remain nearly consistent across the entire temperature range, from liquid nitrogen temperatures to room temperature. This consistency underscores the stability of phonon vibrations in Ta_3_VSe_8_ through the whole temperature regime and suggests a negligible thermal expansivity within the interested experimental conditions. This trait sets it apart from the usual behavior seen in typical TMDs like MoS_2_, WSe_2_, and Ta_2_NiSe_5_, which exhibit distinct temperature-dependent phonon dynamics [59,60,61].

We further proceed with a magneto-transport measurement to uncover the magnetoresistance of Ta_3_VSe_8_. The schematic of our experimental configuration is illustrated in Figure 4a. Silver conductive paint ensured a solid connection of the four silver wires between the sample and the electrodes on the sample holder. This four-probe setup offers the advantage of minimizing the effects of contact resistance, a notable improvement over a simpler two-probe configuration. In our experimental framework, the voltage (*V*_+_ and *V*_−_) detected between the two probes near the middle of the sample gives the sample’s longitudinal resistance. The dependence of this resistance upon an applied magnetic field defines the sample’s magnetoresistance. In this setup, the lock-in amplifier acts both as a voltmeter to capture the longitudinal voltage and as a source to generate a constant voltage signal. This constant voltage (*V*_source_) is then converted into a constant current (*I*_out_) by a voltage-controlled current source, enabling us to calculate the longitudinal resistance ($R_{\mathrm{xx}}$) by the formula $R_{\mathrm{xx}}=\frac{V_{+}-V_{-}}{I_{\mathrm{out}}}$.

The magnetoresistance measurements, spanning a temperature range from 2 K to 160 K, are illustrated in Figure 4b, highlighting the sample’s magnetoresistance at lower temperatures. We also calculate the longitudinal resistance into magnetoresistance (MR) to clarify the variation in resistance clearly in Figure 4c, $\mathrm{MR}=\frac{R_{\mathrm{xx}}\left(B\right)-R_{\mathrm{xx}}\left(0\right)}{R_{\mathrm{xx}}\left(0\right)}\times 100\%$. Our findings indicate that, above 100 K, the variations in magnetoresistance are minimal, remaining essentially within the bounds of the signal-to-noise ratio. As the figure suggests, the sample manifests a slightly positive magnetoresistance behavior as the magnetic field increases from 0 to 7 T. This indicates an increase in electronic scattering events with the strengthening magnetic field. Of additional interest is an observed fluctuation in the zero-field resistance, which differs from a simple monotonic temperature dependence. As the sample temperature climbs (transitioning from red to blue in the plot), the resistance behavior shows an initial decrease, followed by an increase, then subsequently a decrease, and ultimately an increase again. This characteristic diverges from that of MoS_2_, WSe_2_, and Ta_2_NiSe_5_, which exhibit a monotonic temperature-dependence in their resistance [36,62,63]. Previous studies may address this phenomenon on the Lifshitz transition [64,65,66], metal-insulator transition [67], or the exotic metallic state, which shows bad metal behavior [68,69,70] in resistance-temperature curves. The mechanism behind the fluctuation of zero-field resistance remains unclear, and it raises the hypothesis that the material may experience a phase transition as the temperature varies.

## 4. Conclusions

In summary, we have synthesized Ta_3_VSe_8_, a new 2D ternary TMD material, using the CVT method. The resultant single crystals, approximately 9–25 mm^2^ in size, boast a shiny appearance and can be easily exfoliated with terrace features on the sample surface. While the EDX elemental mapping confirms the homogeneity of element distribution, the XRD analysis confirms its structure symmetry and lattice constant, which further supports the single-crystalline nature of the sample. Through temperature-dependent Raman spectroscopy spanning from the liquid nitrogen temperatures to the room temperature, stable intensity and the position of Raman peaks reveal the stability of sample lattice and phonon modes. Moreover, positive magnetoresistance is found at low temperatures and gradually suppressed at higher temperatures. Our work may provide a material platform for growing and researching ternary compound TMD materials.

## Data Availability

The data that support the findings of this study are available upon reasonable request from the authors.
